# Detection of cytological abnormalities in urothelial cells from individuals previously exposed or currently infected with *Schistosoma haematobium*

**DOI:** 10.1371/journal.pone.0278202

**Published:** 2023-03-30

**Authors:** Cecilia Smith-Togobo, Richard Mprah, Evans Adamu Yeboah, Harry Koku Anyidoho, Dzifa Asigbe, John Kwame Afernorfe, Felix Ayroe, Kwabena Obeng Duedu

**Affiliations:** 1 Department of Medical Laboratory Sciences, School of Allied Health Sciences, University of Health and Allied Sciences, Ho, Ghana; 2 Ho Municipal Hospital, Ghana Health Service, Ho, Volta Region, Ghana; 3 Department of Biomedical Sciences, University of Health and Allied Sciences, Ho, Ghana; University of Ibadan, NIGERIA

## Abstract

Urinary schistosomiasis has long been associated with bladder cancer, but it is still not clear the mechanisms involved. *Schistosoma haematobium* causes injury and disruptions in the integrity of the urothelium. The cellular and immunologic responses to the infection lead to the formation of granulomata. The ability to use cellular morphological changes to predict the risk of developing bladder cancer following *S*. *haematobium* infection is thus important. This study assessed the cellular changes in the urine associated with schistosomiasis and the potential of routine urine being used as a risk predictor of the development of bladder cancer. Urine samples (160) were screened for the presence of *S*. *haematobium* ova. Smears stained with the Papanicolaou method were evaluated using light microscopy to determine the cell populations. A high prevalence (39.9%) of urinary schistosomiasis and haematuria (46.9%) was found among the participants. Polymorphonuclear cells, normal and reactive urothelial cells and lymphocytes were characteristic of *S*. *haematobium* infection. Squamous metaplastic cells (SMCs) were found in 48% and 47.1% of participants who have had past or current S. haematobium infection respectively, but were not found in participants who had no exposure to S. haematobium. These squamous metaplastic cells are in transition and are prone to malignant transformation when exposed to a carcinogenic agent. There is still a high burden of schistosomiasis in endemic communities in Ghana. by examining urine, one can find metaplastic cells and? dysplastic cells and thus predict cancer in SH-infested patients. Thus, routine urine cytology as a tool to monitor the risk of bladder cancer development is recommended.

## Introduction

Schistosomiasis is a common infectious disease in many parts of the world especially Africa [1]. Globally, schistosomiasis ranks second after malaria in terms of its rate of incidence and burden of disease [1–4]. Most of the affected areas suffers inadequate sanitation problems [1] and depend on rivers and streams as their source of water. The risk of infection is through contact with freshwater infected with the parasite [5, 6]. There are five species of *Schistosoma* parasites that cause the infection in humans; *Schistosoma haematobium*, *Schistosoma mansoni*, *Schistosoma japonicum*, *Schistosoma intercalatum* and *Schistosoma mekongi*. However, most of the disease burden is associated with *S*. *haematobium* and *S*. *mansoni* [7, 8].

*S*. *haematobium* infection also referred to as urinary schistosomiasis (US) is characterized by bloody urine. The presence of a *Schistosoma* ova is a characteristic pathology in the reproductive organs of females and it is a risk factor for transmission of sexually transmitted diseases (STDs) [1, 9]. *S*. *haematobium* infection can also lead to severe egg-induced pathology and symptoms in the genitals of both men and women [9]. The infection has long been linked to the development of bladder cancer [10–12]. Pathological changes in the bladder due to SH infection have been reported to include squamous metaplasia of the urinary bladder, keratinizing and non-keratinizing metaplasia, and invasive squamous carcinoma [12]. In addition, granulocytes, mixed with normal urothelial and squamous cells inflammatory cells, hyperkeratotic cells and squamous metaplastic cells have also been reported [13, 14]. The importance of urine cytology in cases of *S*. *haematobium* infections have thus been suggested [13, 15].

During the infection, the ova embed in the bladder epithelium (BE) and secrets soluble egg antigen that modulate the immune system resulting in the recruitment of inflammatory cells and secretion of inflammatory mediators eventually leading to the formation of a granuloma around the egg [16–19]. At the same time, the parasite activity in the BE causes its shedding. Chronic or persistent infection by *S*. *haematobium* causes rapid exfoliation of the BE that are later replaced with squamous metaplastic cell SMC [17] which has been associated with the development of bladder carcinoma [20]. However, *S*. *haematobium* infection has been associated with squamous cell carcinoma (SCC) of the bladder for the past decade [19, 21–23]. In a recent review, it was noted that although several carcinogenic pathways have been postulated, the exact mechanism(s) are not yet defined. For example, *Schistosome* cell total antigen has been reported to increase in urothelium cell division with slow death rates. Other studies have also shown that soluble egg antigens (SEA) extracted from Schistosoma egg positive urine enhanced excessive proliferation, increase the oxidative stress (through reduced glutathione (GSH) depletion), and decreased apoptosis in cultured human prostate (PNT2) cells. These and other pathways have been suggested as potential pathways in the development of cancer due to *Schistosome* infection [21].

Most studies carried out on schistosomiasis in Africa focused on the parasite and the epidemiology of SH infections [1, 2, 5, 8, 22, 24–26] with less emphasis placed on the cellular features in the bladder epithelium of exposed individuals that represent a risk for bladder cancer. Therefore, this study sought to identify cellular changes in BE of individuals with *S*. *haematobium* infections which could be associated with the risk of the development of bladder cancer.

## Materials and methods

### Ethical issues

The study protocol was approved by the Research Ethics Committee of the School of Allied Health Sciences, University of Health and Allied Sciences, Ho, Ghana (Ref. No. UHAS-REC A.9 [115] 20–21). Parental consent was obtained from parents of children under 17 years old. In addition, child ascent was obtained for children above 6 years old. For adults written informed consent was obtained after the study procedure was clearly explained to them in local languages or English language.

### Study site

The study was carried out in Mafi-Mepe and Mafi-Devime in the North Tongu and Central Tongu districts respectively of Ghana. Both are rural communities located along the basin of the White Volta (lake). Most natives in these communities are either fishermen or farmers and they rely on the lake as their main source of water for drinking, bathing, washing, cooking, and other chores.

### Study design

A cross-sectional and a purposive sampling method were used in this study. An announcement was made through the town criers in the study communities for natives who are showing any sign of schistosomiasis (blood in urine except those menstruating) or have had the disease before to meet the investigators at the basic school compound.

### Sample collection and processing

A total of 160 urine samples were collected from participants over two (2) months period (March-April) from both adults and children; natives from Mepe and Devime or have stayed in the communities for at least six months and have had exposure to water from the White Volta. The urine was collected into a 50ml sterile, plastic screw capped containers between 8am– 12pm after a questionnaire was administered to them. The samples were taken between these periods to allow maximum exertion that dislodges the parasite for easy shedding during urination. Participants were instructed to catch the last drops of urine as well because the *Schistosoma* parasites ova are mostly shed in the last drop [23].

Samples were kept on ice and transported to the laboratory within three hours after collection. At the laboratory, the macroscopic appearance of each sample was described, including the volume, colour, and consistency. About 2ml of each urine sample was dispensed into a centrifuge tube after a gentle mix and centrifuged at 1500rpm for 5 minutes, after which the supernatant was decanted. The urine sediments were resuspended, and two drops placed on a clean grease-free glass slide using a Pasteur pipette and cover slipped. The wet mount was observed under a light microscope (Olympus CX41RF, Tokyo-Japan) using 10x and 40x objectives for the presence of *S*. *haematobium* ova.

Smears for cytological examination was prepared by putting 2 drops of the resuspended sediments on a labelled slide. Another labelled slide was placed on top of it and pulled apart sharply. The slides were immediately fixed with 95% ethanol for 15 minutes after which they were stained using the Papanicolau’s staining technique. Briefly smears were washed in distilled water for one minute after fixation then stained in Harris haematoxylin for 6 minutes, blued under running tap water for 5 minutes, partially dehydrated in 70% and 95% ethanol for one minute each. After that, the smears were stained in Orange G6 for 2 minutes, rinsed in 2 changes of 95% ethanol for 1 minute each then stained in Eosin-azure for 3 minutes. The smears were dehydrated completely in 2 changes of 95% and absolute ethanol for 2 minutes each, after which they were air-dried at room temperature and mounted with Distreen plasticiser xylene (DPX) and observed under a light microscope (Olympus CX41RF, Tokyo-Japan) for cytological evaluation using 10x and 40x objectives. The following features were considered for the cytological evaluation: general morphology of the urothelial cells, nuclear size, shape and colour of the cells, cellular arrangement, the presence of other cellular features associated with the cells and the background of the cells (inflammatory cells). A smear was said to be positive for the presence of any of the cells when they were >10 per high power field.

### Data analysis

Statistical analysis was carried out using Statistical Package for Social Sciences (SPSS) Version 22 (IBM). A descriptive analysis of the sample was conducted, according to the presence of haematuria and considering the distribution of gender, village, age and treatment. Results were expressed in frequencies and percentages and presented as tables and graphs. Fisher Exact test was used to determine the attributable risk of the exposure to *S*. *haematobium* and the cellular composition of the urine. P-value < 0.05 was considered as statistically significant.

## Results

### Characteristics of study participants

A total of 160 participants were recruited for the study, 65 from Devime and 95 from Mepe. 91 (56.9%) of the participants were males and 69 (43.1%) were females. Study participants were between the ages of 6–35 years with majority falling within the ages of 11–20 (12.5%), followed by 16–20 years (6.9%) and finally ages ≤10 (1.9%) with a median age of 16.05 years. Most of the participants (98.8%) were students. Most respondents (51.9%) were within the age ranges of 16–20 years, followed by 11–15 (38.8%). Majority (98%) of respondents were students with the level of education for all respondents being basic as at the time of the study.

#### Distribution of symptoms experienced by respondents

Seventy-five (75 of participant had *haematuria*, 51.9% did not show any sign and symptoms of the disease and 1.3% had itching in their genitals. 42.5% of participants had received treatment and more than half (57.5%) had never received treatment for the disease. Of the 95 recruited from Mepe, 67.4% had haematuria, 30.5% had no haematuria and 2.1% of the 95 had genital itching. Out of the 65 recruited from Devime, 16.9% had *haematuria*. Over half (52.3%) of respondents from Devime had received treatment for SH prior to the study. No participant from Devime tested positive. Of the 95 recruited from Mepe, more than half of the respondents (67.4%) had haematuria and had never received treatment for SH, and 2.1% had genital itching 35.8% had received treatment as at the time of the study.

Out of the total number recruited, 126 produced urine which was screened for urinary schistosomiasis. Of these 27% (34) were positive for *S*. *haematobium* ova.

### Cellular changes in the bladder epithelium with or without *S*. *haematobium*

**Fig 1:**
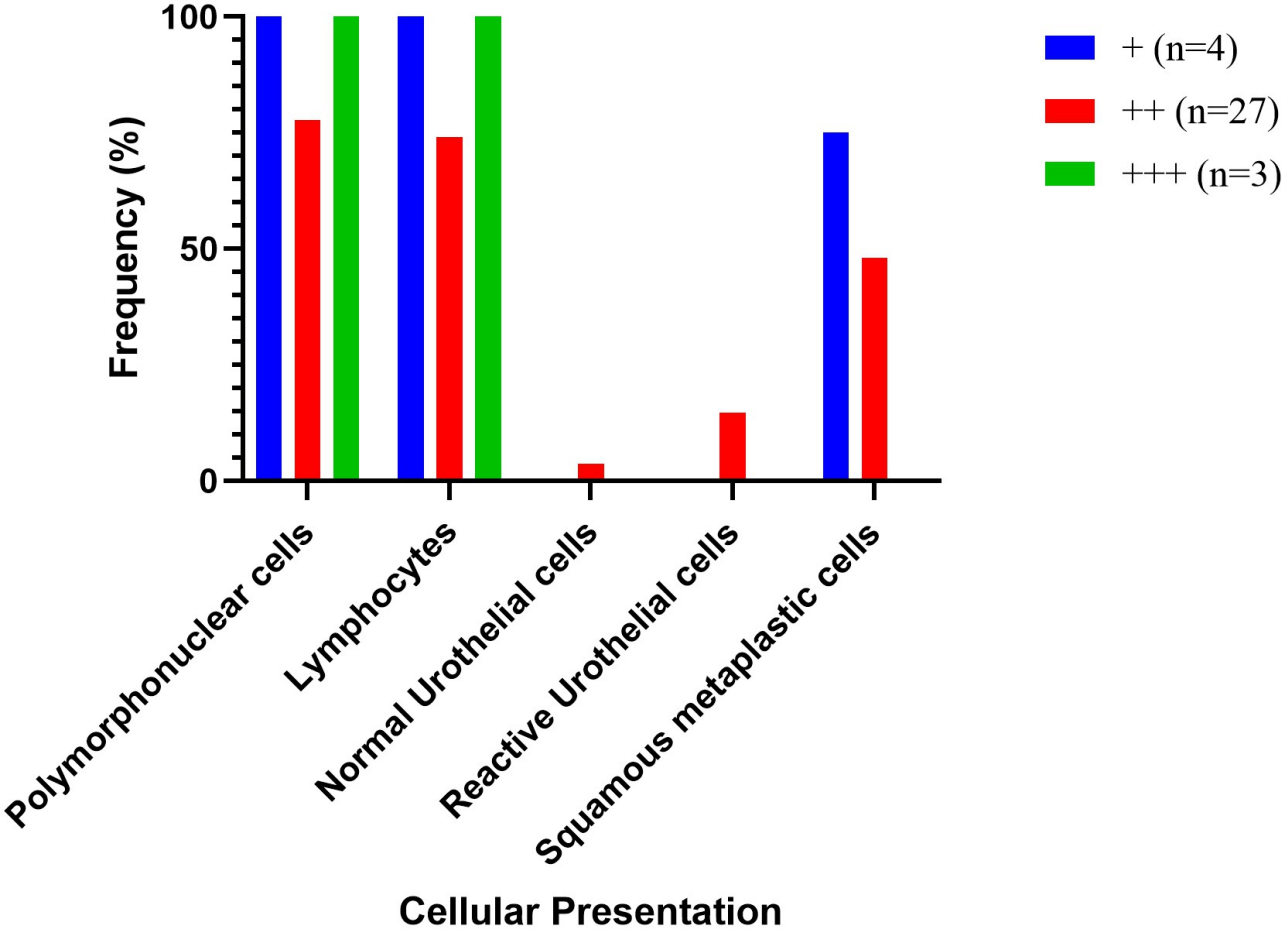
Cellular changes in *S*. *haematobium* positive urine samples. Grading of *S*. *haematobium* positivity: + (<5 *S*. *haematobium* ova per high power field), ++ (5–10 ova per high power field), +++ (>10 ova per high power field).

Polymorphonuclear cells (PMNs) were seen in majority (82.4%) of the samples whereas lymphocytes were seen in 79.4% of the *S*. *haematobium* positive smears (Fig 1). Sixty-eight urine samples were negative for *S*. *haematobium* ova, of which 36.8% were from individuals who had been exposed to the parasite in the past. Of the exposed individuals, 52.0% had lymphocytes, 44.0% had PMNs. Squamous metaplastic cells (SMCs) were not found in participants who had no exposure to *S*. *haematobium*. On the other hand, SMCs were found in 48% and 47.1% of participants who have had past or current infection with *S*. *haematobium*, respectively. The attributable risk of *S*. *haematobium infection* leading to the occurrence of SMCs in individuals with past or present infection was 48.0% (95% CI of 25.8–68.25%) and 47.1% (05% CI of 27.31–64.60%) respectively (Table 1). However, 24 of the smears were acellular and were excluded from further analysis.

**Table 1:** Cellular changes in the smears of SH negative urine samples.

Parameter	*S*. *haematobium*		Total	Attributable risk	P-value*
	*Not-Exposed*	*Exposed*			
**Total**	**43**	**25**	**68**		
**Squamous cell**					
Not seen	39(90.7%)	0(0.0%)	39(57.4%)	90.7 (95% CI 69.2 to 96.9)	<0.0001
Seen	4(9.3%)	25(100.0%)	29(42.6%)		
**Squamous Metaplastic cell**					
Not seen	43(100.0%)	13(52.0%)	56(82.4%)	48% (95% CI 25.8 to 68.3	<0.0001
Seen	0(0.0%)	12(48.0%)	12(17.6%)		
**Polymorphonuclear cells**					
Not seen	38(88.4%)	14(56.0%)	52(76.5%)	32.4% (95% CI 8.6 to 54.3)	<0.0062
Seen	5(11.6%)	11(44.0%)	16(23.5%)		
**Lymphocyte**					
Not seen	43(100.0%)	12(48.0%)	55(80.9%)	52% (95% CI 29.3 to 71.67)	<0.0001
Seen	0(0.0%)	13(52.0%)	13(19.1%)		
**Urothelial**					
Not seen	31(72.1%)	13(52.0%)	44(64.7%)	20% (95% CI -5.3 to 43.7)	0.1183
Seen (normal)	12(27.9%)	10(40.0%)	22(32.4%)		
Seen (Reactive)	0(0.0%)	2(8.0%)	2(2.9%)		

*P values reported are for the fisher’s exact test which was performed

## Discussion

Urinary schistosomiasis has long been associated with bladder cancer but it is still not clear the mechanisms involved. People infected with *S*. *haematobium* stand the risk of developing bladder cancer in future [19]. The morphological changes in the bladder epithelium can predict activity and injury to the bladder because of *S*. *haematobium* infection. This study is one of very few studies that looks at cellular changes in the bladder epithelium due to *S*. *haematobium* infection in Ghana and globally. About half of the respondents in this study acknowledged having received praziquantel as treatment for *S*. *haematobium* infection. This is not surprising as the communities studied are known to be endemic for the disease [25, 26] and there are mass drug administration (MDA) programmes that have been implemented [27]. The relatively high prevalence of *S*. *haematobium* infection however is worrying and raises a question as to the effectiveness of the interventions that have been made so far. Furthermore, it is also noted that although some individuals tested negative for the presence of eggs in the urine, they reported haematuria, a classical symptom of the infection. This could suggest very low levels of infection where the parasites may be few in numbers. This suggests there is still a need for public health enlightenment in areas endemic for *S*. *haematobium* in the Volta basin, Ghana.

Most of the *S*. *haematobium* positive samples had PMNs and lymphocytes which is a sign of inflammation. This could be due to bladder injury and disruptions to the urothelium. This is consistent with a recent report that described a case of urinary schistosomiasis where histopathology revealed male and female worms in the bladder together with active granulomas and intense inflammatory cells infiltration consisting mainly of polymorphonuclear cells and lymphocytes [16]. The presence of lymphocytes is also characteristic of the formation of granulomas due to the *S*. *haematobium* infection. The granuloma forms around the eggs of parasite and prevent their release into the bladder and subsequent excretion [16, 28, 29]. In addition to PMNs and lymphocytes there was also an increase in normal and reactive urothelial cells as well as squamous cells. In mice models, it was found that *S*. *haematobium* eggs in the bladder led to urothelial hyperplasia, a potentially pre-cancerous lesion [30]. The presence of products not found in normal urine but in the urine of *S*. *haematobium* infected individuals is not unusual. In an analysis of the human urine metabolome, it was found that urine containing *S*. *haematobium* eggs had several products including mutation and carcinogenic precursors like estrogen-like metabolites and guanine-derived oxidation products not found in normal urine [31–33]. Furthermore, the increase in urothelial cells can be attributed to the destructive activities of *S*. *haematobium* worms in the bladder epithelium and the ability of the worms to induce proliferation of the bladder epithelium.

The presence of normal squamous cells is usually considered as contamination but the presence of SMC indicates that there is some form of repair to replace cells that have shed from the bladder epithelium most likely due to the activity of the worms. SMC are transitional cells that develop to replace the normal epithelium in abnormal conditions like *S*. *haematobium* infections [34]. Although it is unclear the clinical significance of the SMCs, studies suggest that these cells are associated with the development of squamous cell carcinoma [35]. In this study we found about half of the participants who had either had past infection with *S*. *haematobium* or had a present infection had SMCs in their urine. Strikingly, those who had not been exposed to the parasite did not have SMCs in their urine, strongly suggesting a close link between the parasite and the development of SMCs. The SMCs in urine have been associated with response to chronic inflammation of the bladder especially the trigonal area, that can be induced by the deposition of the *S*. *haematobium* ova in the bladder epithelium and possibly continuous exposure to the parasite [35]. When the infection persist or re-infections occur there is the tendency of the SMC to develop into Keratinizing SMC, increasing the risk of bladder cancer [10, 36]. Likewise, when SMC undergo malignant transformation upon exposure to cancer causing agents like human papilloma virus (HPV) [1, 10], the risk of developing cancer of the bladder is increased. Though there is no specific age at which infestation occur, it has been observed that children at an early stage of life who tend to swim in or drink an infested water become infected with the parasite. With the sequence of event from infection to the development of cancer, children who get frequent infections are prone to developing bladder cancer in an older age. In this study a an average age of 16 years testing positive for the parasite with some showing squamous metaplastic cells in their urine, such individuals living in the same area for a long time with no interventions in place, the chance of frequent re-infection is high, and therefore the risk of developing bladder cancer in their old age [31].

## Conclusions

Our study shows that there is still a high burden of *S*. *haematobium* infection in the endemic communities despite the interventions made. Furthermore, there is a strong linkage of *S*. *haematobium* infection to the transition of the bladder epithelium evidenced by the presence of SMCs. Urothelial cells, PMNs and lymphocytes were also found to be closely linked to *S*. *haematobium* infection. These findings throw more light on the role of *S*. *haematobium* in the development of bladder cancer. We therefore recommend that all *S*. *haematobium* infected patient be monitored by routine urine cytology to help administer appropriate interventions. A longitudinal study to establish the causality of SMC and the development of bladder cancer is needed.
